# The Institutional Worker

**Published:** 1912-12-14

**Authors:** 


THE
### INSTITUTIONAL WORKER
Being a Special Section to "The Hospital"

### BUREAU OF INFORMATION.

### Rules for Correspondents.

1. Every letter, on whatever subject, must be accompanied by the coupon to be cut from the back cover (inside page) of The Hospital, current issue, and must contain the name and address of the correspondent with pseudonym for publication if desired.

2. Replies by post cannot be given, save under exceptional circumstances at the Editor's discretion.

3. Letters from Approved Homes in reply to special needs published in the Bureau should state terms and full particulars, and be sent prepaid under cover to the Editor of the Bureau with name written across coupon for identification.

4. Proprietors of Homes which have not yet been entered on the List of Approved Homes, but have spare accommodation likely to suit special needs, are invited to write for an application form for registration. The fee for registration, which includes two announcements of the Home in the Bureau, is 5s.

5. Special needs of every description relating to those who are sick or in difficulties are made known in these columns free of charge.

6. All communications to be addressed to The Editor of The Hospital, 28 Southampton Street, Strand, London, W.C., and marked "Bureau of Information."

### Approved Homes.

This column is reserved strictly for announcements of Homes that are registered on the "Approved Homes List" of The Hospital Bureau of Information (see p. 1 of this section), and no Home is added to the list without direct medical and other testimony of its good management.

**Clacton-on-Sea.**—Melrose Nursing and Convalescent Home, Marine Parade. Principal: Miss Kate Heathcote. Medical, surgical, maternity, and convalescent cases. Special advantages for children. South aspect, with broad verandah facing sea.

**Cheshire.**—One little girl can be received for education with principal's own child in pleasantly situated house in the country. Skilled nursing and healthy open-air life for delicate child. Address K. W. (No. 52), c/o The Bureau.

### Training and Employment.

**Training for District Work.**—The offer of a vacancy at the excellent provincial hospital you name is, in our judgment, too valuable to be refused. You might have to wait a long time before obtaining a vacancy in London, and the strain of work would be very much greater. Write, however, first to the General Superintendent, Queen Victoria's Jubilee Institute for Nurses, 58 Victoria Street, S.W., informing her of your desire to be trained for this branch of work and take her advice. We do not think that you would find any opposition would be made in any hospital to your attending Mass, but you would, of course, have to take your turn with others in respect of Sunday privileges.—D. M. J.

### Needs and Helpers.

**Home Found.**—We are greatly indebted to you for so kindly receiving at a low charge the consumptive girl about to be discharged from Brompton. The other lady of whom we wrote to you is much better and able to undertake light employment.—Spes.

**Nursing Aid for Consumptive.**—We note that your father is lying in the last stage of consumption, suffering acutely from bed-sores, with only your inexperienced sister to attend upon him. We have much sympathy with you in this anxiety, and have communicated the circumstances to the Acland District Nurses' Home at Oxford. There is hardly any town where cases of tuberculosis are more diligently sought out and attended to than at Oxford, and we have no doubt immediate relief and assistance can be procured.—Ethel W.

**Home Employment for Widow.**—We fear there is no hope of your obtaining envelopes to direct or any other home work of that description. Nor can we recommend you to take a house with a view to taking in boarders. If you will write and tell us your age, your late husband's occupation and that of your son, and what work, if any, you have been accustomed to do we will advise you further.—Anne T.

**Sanatorium Treatment at Low Terms.**—We are glad you are turning your attention to meeting this great want. If you can make it cover expenses by charging 15s. a week to adults and 8s. a week for children you will be very clever. Should you carry out your plan, write to the Invalid Children's Aid Association, 69 Denison House, Vauxhall Bridge Road, London, S.W., and to the almoners of the large London hospitals, who are often in need of this kind of accommodation. Best wishes.—M. R.

### Nursing in the Empire and Abroad.

**Nurses for Suez.**—We are much disappointed that you and your friend have decided against this post after all. The opening is a very good one, and we are anxious to hear of a fully-qualified maternity nurse to take up work there early in the year. Do you know of anyone else?—Gertrude.

**Life in Valparaiso.**—The profession of nursing appears to be entirely without organisation in South America. Hence a nurse settling here would have all to do in making her way and actually rousing a desire for trained nursing. As a natural consequence fees are low, while the cost of living is high. Valparaiso is a purely commercial centre, and though there are many rich residents, the majority are traders without much money to spend on the "luxury" of being nursed. Spanish is the language of the country. Next year an English hospital is to be opened, and should you desire, for private reasons, to practise your profession in this locality you should endeavour to obtain an appointment on the staff. Let us know if you would like an introduction.—Pioneer.

**Conditions at Port Elizabeth.**—As usual in Cape Colony, Colonial nurses are preferred here to Englishwomen. We cannot recommend you to risk the inevitable disappointment of trying to make a nursing connection in the teeth of strong local competition. Fees £3 3s. to £5 5s. a week. There are many formalities to go through before taking up work under the State regulations.—Amice.

### Letter-Box.

**The Editor begs to acknowledge the receipt of communications, with enclosures, from:**—The Misses C. H., Teignmouth.

**Communications have been received and attended to from:**—Mr. A. Stevens; Miss B. E., Dereham; Miss S. A. W., Droitwich; Sister E. M. E., Hemel Hempstead; Miss K. H., Clacton; Miss G., Worthing; Miss C. B., Cork; Mrs. L. H. H., Hendon; Mrs. S., Ampthill;

Mrs. T., Bristol; Nurse C. B., Liskeard; Nurse H., Bexhill-on-Sea; Miss MacG., Hatherleigh; Miss C., South Kensington.

**The Editor is much indebted for testimonials in response to inquiries from:**—The Charity Organisation Society; Dr. Rogers; Dr. Blake.

**Letters have been forwarded from:** — Mr. M. E. A. M., Miss A. H., Mrs. W., Miss G., Sister A., Miss M. R., Mrs. D., and others.

**The Editor is unable to introduce patients to any but the Homes on our Approved List:**—Mrs. B. H., Easingwold; Miss T. H., Worthing.

We note that no more applications are needed for this case.—F. P. (136a).

We note that there are no trained nurses in Colombia, and that it is preferable they should not come.—Acting British Vice-Consul, Bogota, Colombia.

Very pleased to learn that you have been able to make such a comfortable arrangement for your friend at one of our Homes.—K. M. L. (135a).

There is no occasion for names and addresses to be published.—Mrs. W. Cheshire.

Many thanks. We note that Swedish-trained nurses who speak English can generally be obtained if required.—H.M. Consul, Stockholm.

Your communication received with thanks. — British Vice-Consul, Granada; H.M. Vice-Consul, Caracas.

We recommend you to advertise your bound volumes of The Hospital in The Hospital and the Sale and Exchange Columns of *The Nursing Mirror*.—Miss G.

**Persons requiring nurses and nurses requiring appointments will find their needs met in the adjoining pages, where many useful announcements appear this week.**

### Housekeeping Queries.

**To Preserve Table Knives.**—You must remember that it is not use which destroys your knives, but careless handling in the kitchen or treatment in a knife-cleaning machine which is out of order. If the knives are getting worn at the top, suspect the machine first and have it seen to. Then follow this routine: The knives should be removed from the dining-room in a special knife-tray, and should be placed at once in a jug with hot water and soda, which should cover the blades only, to prevent the handles becoming loosened. They should be thoroughly well washed, dried on an old cloth, cleaned on a board or in a good machine, dusted, and placed in a wooden box, keeping large and small knives separate. Knives should never be left dirty, but be wiped, at least, at once to prevent stains on the steel. Stains on the white handle can usually be removed with lemon-juice and French chalk.—E. J. H.

**Salads.**—We quite agree these should be a daily feature of the menu, but they need not be as costly as you imagine. By utilising any cold vegetables left over to mix into the salad a good variety can be had at little cost. Cold cabbage chopped up with celery and a suspicion of onion, and mixed with a good mayonnaise sauce, makes a most excellent salad; it can be used up in that way with more success than when served hot. Cold butter beans or haricot beans can be made into excellent salads as well. Brussels sprouts can be utilised too, but care must be taken to cut away the white centre, as that has a very strong flavour. A little grated cheese sprinkled over this salad is an improvement if cheese is liked.—Fanny P.

### Institution Facts and Figures.

### AN INVITATION TO THE PRACTICAL WORKER.

In every well managed institution there is an accumulation of valuable observations, extending often over a term of years, which are wasted because there is no medium through which they can be brought to the notice of fellow-workers in the same field. It is our hope through this section of the "Institutional Worker" to collect some of these facts and present them to our readers. Every month two questions will be set relating to the internal working of institutional life, its cost, and distinctive features. The best answers will be published in due course, and payment will be made for contributions at the rate of Five Shillings for each answer which the Editor considers of sufficient interest for publication.

Questions for December.

1. What was the exact amount of milk consumed in a given week in December in your institution (*a*) in a medical ward; (*b*) in a surgical ward? State daily average of patients. What was the average cost per head of each patient in those wards respectively for milk during that week calculated on the contract price?

2. What is the most popular supper dish (savoury) in your institution? Give the recipe and quantity of each ingredient required for fifty persons, and state the cost per head.

The figures published by King Edward's Hospital Fund relating to the cost of various articles in London hospitals have brought forcibly before the public the variations which occur in the cost of everyday articles in London institutions. If our readers will help us we hope to throw additional light on the hospital commissariat throughout the kingdom.

Answers are invited from workers in every class of institution for the sick, and the value of those published will be greatly enhanced if opportunity be afforded for contrasting figures in various parts of the kingdom. The name of the institution need not be mentioned unless desired. The only condition is that the figures must be the result of actual observation and computation, not estimates. Either or both of the questions may be answered by each contributor, and payment will be made for all answers published as above mentioned.

The following rules must be observed by contributors to the Facts and Figures Column:—

1. Answers must be written on one side of the paper and must bear the name and address of the sender and be accompanied by coupon to be cut from the back cover (inside page) of the current issue of The Hospital. A pseudonym must be chosen if the name is not to be published.

2. Answers must be addressed to the Editor of The Hospital, 28-29 Southampton Street, Strand, London, W.C.; they must be marked in the left-hand corner "Facts and Figures," and must be received before the last day of the current month.

### Editor's Notices.

**Contributions:**

Contributions should be written, or preferably typed, on one side of the paper only, and all articles sent in are accepted upon the distinct understanding that they are forwarded to The Hospital only.

The Editor cannot undertake to return MSS. not used, but when a stamped directed envelope is enclosed the MSS. may be returned if a special request is made.

Accepted articles and paragraphs of news will be paid for after publication at the scale rate.

**Address:**

To prevent delay all contributions and letters on editorial business must be addressed exclusively to the Editor, "The Hospital," 29 Southampton Street, Strand, London, W.C. It is important that this regulation shall be strictly observed.

**Correspondence:**

Correspondence on all subjects is invited. The name and address of correspondents must be given as a guarantee of good faith, but not necessarily for publication.

**Special Articles:**

Special articles are invited, and questions, inquiries, and paragraphs upon all matters relating to the work, administration, and management of general, special, mental, fever, cottage and convalescent hospitals, sanatoria, homes, institutions, societies and organisations for the treatment or care of the sick, injured and dependants of all classes. Every matter affecting the interests and welfare of the staff of all grades working in these institutions will receive special consideration.

**Administrative Medicine, Research and Items of News:**

Special terms will be paid for approved articles on subjects in this wide field by experts who have made some section of it their special study and interest. We wish to give prominence to every movement tending to advance accuracy of diagnosis, efficiency in treatment, and the development of modern methods to eradicate disease and promote the welfare of the suffering.

**A Bureau of Information:**

We invite inquiries and applications for information and help in providing for that numerous class of sufferers who require special care or house-room which they cannot obtain in their own homes or secure for themselves. **We cannot, however, prescribe, or recommend practitioners.**

**Books for Review:**

Publishers are particularly requested to send advance proofs of any new books of importance whenever possible, as the Editor has made arrangements to publish immediate reviews on a new plan.

**Photographs, Plans, Blocks, and Illustrations:**

It is requested that wherever possible MSS. may be accompanied by illustrations in any of the above forms. The name of the sender and of the article to which it belongs should be written on the back of each photograph, drawing, or block for purposes of identification.

**Local Papers:**

Newspapers containing reports or news paragraphs should be marked and addressed to the Sub-Editor.

**The Coupon System:**

A coupon will be found at the bottom of the third inside page of the cover each week. This coupon must be attached to every question or inquiry to which an answer is desired in The Hospital.

### When Seeking an Appointment.

If you are desirous of securing a post in any department of the Institutional, Health, and Sanitary services, The Hospital affords an exclusive and successful medium of direct approach to the Governing Bodies and Executive Officials of Hospitals, Poor-Law Infirmaries, Asylums, and Institutions of every description throughout the United Kingdom, in addition to those responsible for the Administration of Public and Municipal affairs.

It is an indisputable fact that in seeking an appointment success is largely dependent upon the choice of a suitable channel for giving adequate publicity to your capabilities and requirements.

"The Institutional Worker" Supplement is especially designed to enable workers to bring their needs prominently to the notice of those in authority, and a column is reserved for announcements of "Appointments Wanted," in which an advertisement of 21 words can be inserted for the nominal charge of 1s.

If you are seeking re-engagement or desire a change you should at once avail yourself of the exceptional facilities which "The Institutional Worker" provides. It has repeatedly proved itself a valuable help to others in similar circumstances, and you would be well advised to employ *it always* whenever the occasion arises.

### Manager's Notices.

**All letters on business matters must be addressed to The Manager, "The Hospital," 28 and 29 Southampton Street, London, W.C.**

**Advertisements:**

To ensure insertion, all advertisements for The Hospital must reach the Manager not later than Wednesday at noon in each week. For scale of charges see pages vii and 4.

**Subscriptions,** which may commence at any time, are payable in advance. Cheques and Post-Office Orders should be crossed London County and Westminster Bank, Covent Garden Branch, and made payable to the Manager of The Hospital.

Rates of Subscription (including Postage).

| *United Kingdom.* | | *Foreign and Colonial.* | |
|---|---|---|---|
| Three Months ... ... | 2s. 0d. | Three Months ... ... | 2s. 6d. |
| Six Months ... ... | 4s 0d | Six Months ... ... | 5s. 0d |
| Twelve Months ... ... | 6s 6d | Twelve Months ... ... | 8s. 8d |

*SUBSCRIPTION FORM.*

*Please send me* The Hospital *for..............months, commencing with your issue of............................, for which please find enclosed...........................*

*Name*...............................................

*Address* ...............................................

...............................................

*Date*....................................

*To* ................................ *Newsagent.*

Or to

*The Manager,*
The Hospital,
The Hospital Building,
28/29 Southampton Street,
Strand, London, W.C.

**Remittances:**

**It is especially requested that remittances be made by Cheque or Postal Order, and in halfpenny stamps only when the amount is under sixpence.**

**THE SCIENTIFIC PRESS, Ltd., 28 & 29 Southampton Street, Strand, LONDON, W.C.**

### HOSPITAL EXPENDITURE: THE COMMISSARIAT.

A USEFUL HANDBOOK FOR HOSPITAL MANAGERS AND MATRONS.

Crown 8vo, bound in cloth boards, **2s. 6d.** post free. Of all Booksellers, or of the Publishers—

THE SCIENTIFIC PRESS, LIMITED, 28/29 Southampton St., Strand, London, W.C.

### BOOKS FOR INSTITUTIONAL WORKERS.

| | |
|---|---|
| Burdett's Hospitals and Charities | 10s. 6d. |
| Hospital Expenditure: The Commissariat | 2s. 6d. |
| Modern Nursing in Hospital and Home | 2s. 6d. |
| Surgical Instruments and Appliances used in Operations | 1s. 8d. |
| Midwife's Pocket Dictionary | 1s. 1d. |
| Surgical Ward Work and Nursing | 5s. 5d. |

Ledgers, Account Books, and every kind of Institutional literature.

Of all Booksellers, or of The Scientific Press, Ltd., 28 & 29 Southampton Street, Strand, London, W.C.

